# Using DosiVox to Reconstruct Radiation Transport through Complex Archaeological Environments

**DOI:** 10.3390/mps2040091

**Published:** 2019-12-11

**Authors:** Amber G.E. Hood, Edmund G. Highcock

**Affiliations:** 1 Department of Geology, Lund University, 223 62 Lund, Sweden; 2 Research & Development, Greenbyte, Östra Hamngaten 16, 411 06 Gothenburg, Sweden; edmund@greenbyte.com

**Keywords:** optically stimulated luminescence dating, ceramics, DosiVox, radiation dose modelling, environmental dose rate, complex burial environments, Egyptian archaeology

## Abstract

The DosiVox programme is used to reconstruct radiation transport in complex depositional environments. Using two archaeological case studies from ancient Egypt, the burial environments for a selection of ceramic vessels are reconstructed using the DosiVox programme, allowing the simulation of the emission and transport of radiation throughout these burial environments. From this simulation we can extract the external dose rate of the archaeological samples, a measurement necessary for determine a luminescence age. We describe in detail how DosiVox can be used to best advantage at sites with complex depositional histories and highlight that DosiVox is a valuable tool in luminescence dating. This work illustrates that DosiVox is, at present, unparalleled in reconstructing a more accurate and detailed external gamma dose rate which can significantly improve upon simplistic scaled geometric models.

## 1. Introduction

### 1.1. Overview

Luminescence dating, including optically stimulated luminescence (OSL) dating, is a valuable tool to archaeologists and geologists and remains arguably the best technique for the chronometric dating of archaeological or geological materials comprised of quartz and feldspar. In this manner, in archaeology, luminescence dating is widely applicable to pottery, minerogenic building materials and ceramic objects or sediments from archaeological contexts. While dating of recently excavated material can be routine (as in situ sampling can be done by a trained luminescence specialist aware of precisely what samples and measurements are required to achieve a luminescence age), working with more complex material, such as that recovered from museum contexts, can be more difficult. While working with recently recovered material suitable for OSL sampling is of course ideal, it is not always possible and in many cases OSL dating is not permitted, owing to strict archaeological laws which coincide with a lack of accessible laboratory facilities.

Archaeological materials housed in museums—most notably pottery vessels and ceramic objects—were often not collected with OSL dating in mind and in some cases were excavated and placed in collections well before OSL dating was even developed. As such, external dose rate (${\dot{D}}_{ext}$) calculations can be incredibly difficult to reconstruct for museum material. Sometimes, for example if no external sediment from the original burial environment is forthcoming, it can be impossible to accurately reconstruct. However in some circumstances we can find museum material which is more favourable to OSL dating, for example, when original depositional sediment is attached to the vessels or is associated in some other way with the material. However, even then, the assumption of a uniform burial environment in archaeological luminescence dating is one that has often proved difficult for archaeologists to reconcile.

With the recent development of the DosiVox radiation transport modelling software [1], it has now become possible to reconstruct radiation transport throughout complex burial environments, where one must consider natural radiation from other archaeological materials, associated geological materials, and structural elements of the environment. Such modelling can vastly improve the accuracy of ${\dot{D}}_{ext}$ determinations for both museum specimens, as well as for complex burial environments recently excavated.

In this paper, we outline the general process for building a DosiVox model of an archaeological site or other complex depositional environments. This model, along with the DosiVox software, allows us to calculate ${\dot{D}}_{ext}$ from first principles and certain basic assumptions, using all the information we have at our disposal (assuming direct measurement is not possible). Thus, for complex depositional environments where direct measurement is not possible, the model represents the best possible estimate of ${\dot{D}}_{ext}$ using tools available at the time of writing.

In Section 1.2 we describe our approach to the measurement of ${\dot{D}}_{ext}$ starting from first principles. In Section 2 we begin by describing the application of the model to two complex sites from Early Dynastic Egypt where direct measurement of ${\dot{D}}_{ext}$ is not possible. We then, continuing into Section 3, compare the results to other possible methodologies for estimating ${\dot{D}}_{ext}$. In Section 4.1 we describe how to construct a DosiVox model based on known information about a site and in Section 4.2 we describe our approach to running DosiVox, including ensuring the quality of the simulations.

### 1.2. Theoretical Background

In our work, we start by observing that, owing to their very short attenuation depth, neither alpha nor beta radiation originating externally to the sample contribute to the equivalent dose. In other words, we assume ${\dot{D}}_{ext}$ is entirely composed of gamma radiation.

With that assumption, three key questions need to be answered when modelling ${\dot{D}}_{ext}$:

1. what was the quantity (flux) of gamma radiation arriving at the target site (the location of the sample) per unit of time;
2. what fraction of that radiation was absorbed;
3. what dose of laboratory-delivered beta radiation would be equivalent to the absorbed dose of gamma radiation?

#### 1.2.1. Grain Absorption and Equivalent Beta Dose

We begin by tackling the second and third questions, which we will find, in our case, to be less interesting than the first. To estimate the fraction of incident radiation that is absorbed by a grain of quartz, it would be possible to model the grains themselves using DosiVox. However, given the extreme difference between the grain size and the gamma attenuation depth (roughly 1:2500) it is highly accurate to model the grains as thin targets, as is the usual practice [2]. This means that we assume that grains are irradiated evenly. Thus, we omit grain models and use DosiVox to calculate the average absorption in a uniform material of the right composition, as we will describe below.

In answer to the third question, we note first that the relationship between the luminescence caused by a dose of gamma radiation and the luminescence caused by the same dose of beta radiation remains impossible to determine from first principles. It would be possible to measure it experimentally by irradiating first with a gamma and then a beta source for the samples in question; however, it is in fact well established that the relationship between the two does not vary from one sample to another, and that it is accurate to assume that they are equal (Reference [3]: 47).

Thus in answer to the second and third questions, it is both standard practice and accurate to assume that the dose is evenly absorbed and the equivalent beta dose is equal to the gamma dose.

#### 1.2.2. Incident Dose

Answering the first question will be the primary focus of this work. In general, the radiation incident on the grains can be directly measured using in situ gamma spectrometry, estimated using the infinite matrix assumption (IMA, see below), or, as in this work, calculated using DosiVox. We begin by briefly discussing gamma spectrometry, then examine in more detail the advantages and pitfalls of the IMA, and then lay out the new possibilities when using a first-principles method like DosiVox.

##### Gamma Spectrometry

Gamma spectrometry is the direct measurement of the incident gamma flux at the site using a portable measuring device, that is, a gamma spectrometer (or a dosimeter, a gamma spectrometer may require an additional assumption about secular equilibrium). If it is possible to take a gamma spectrometer or dosimeter to the sample site and measure the incident dose directly, then that is undoubtedly the easiest method to pursue. However, there are reasons why this may not possible, for example:

- A portable gamma spectrometer is unavailable or cannot be transported to the site.
- The site has radically altered since antiquity; thus, the modern measurement of the gamma radiation intensity may not reflect what it was in the past.
- The context of the sample has already been changed during the current excavation, for example as a result of the removal of stratigraphic layers above.
- The artefact of interest was excavated in the past and its find spot either cannot be accessed or has been destroyed, for example, by backfilling.

##### Infinite Matrix Assumption Plus Geometric Model

Let us now turn to the case where direct in situ measurement of the incident gamma flux is not possible. A second method of determining the gamma flux begins by recovering samples of all materials within the gamma attenuation depth of the sample and determining the intensity of gamma radiation emitted by these materials. This may be through direct measurement using a lab-based gamma spectrometer, or by determining the radioisotopic concentrations within each sample using a form of mass spectrometery (e.g., ICP-MS). The gamma emissions of all commonly discovered radioisotopes are well-measured and readily available [4]. Thus, if we know the radioisotopic content of any material or material mix we know the amount of radiation emitted by that material (per unit mass). Regardless of which method is used, the amount of radiation emitted by each material can be determined.

This is a good start, but what we want to know is the amount of radiation absorbed by the sample (per unit mass). Happily there is one simple case in which one is equal to the other: the case of a homogeneous material of infinite extent. In which case, by symmetry all points in the material must absorb the same radiative energy, and by the conservation of energy, this absorbed amount must be equal to the emitted amount, which we know (given the elemental breakdown). In the case where the material is not infinite, but is nonetheless considerably larger in extent than the attenuation depth of the gamma radiation (typically ∼30 cm in archaeological and geological contexts), we may treat the material as being infinite, and give the absorbed dose as being the emitted dose: this is known as the *infinite matrix assumption* (Ref. [5]: 56).

**Figure 1:**
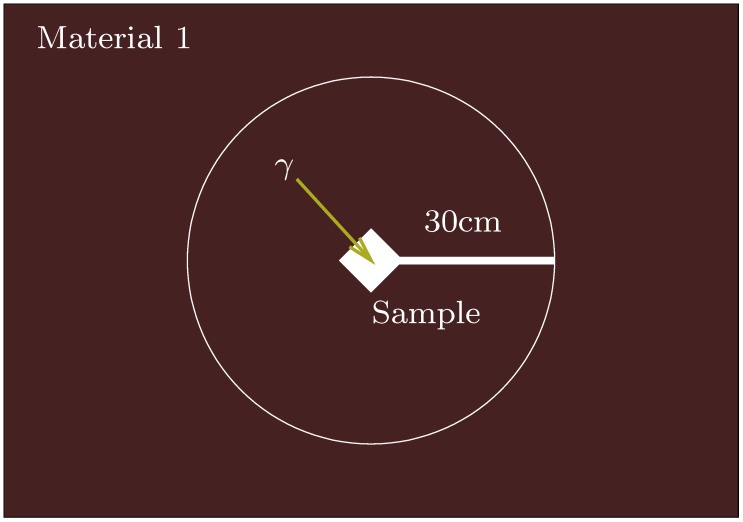
A sample in a simple context for which the infinite matrix assumption (IMA) is valid.

**Figure 2:**
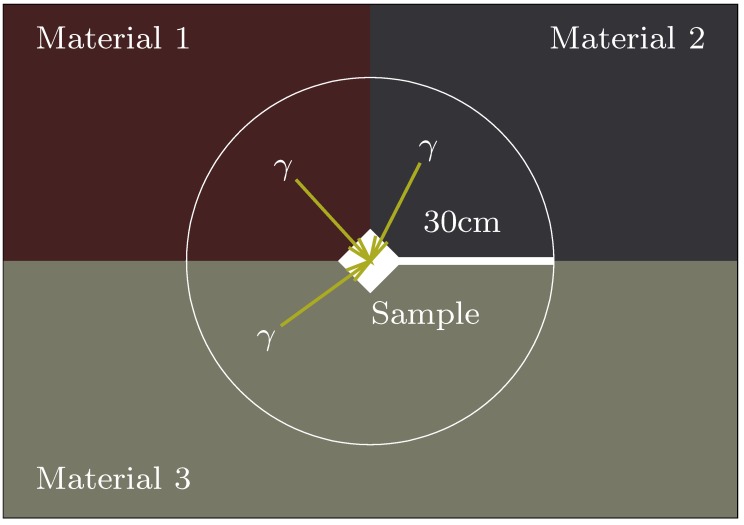
A sample in a context for which the infinite matrix assumption (IMA) in combination with geometric scaling is valid.

The IMA is an excellent assumption if the sample is indeed embedded in a uniform material, as shown in Figure 1, but what if instead it is located at the boundary between two or more materials? Provided the materials can be reasonably approximated as occupying solid angles surrounding the sample, all radiation arriving at the sample only passes through one material before reaching the sample and the infinite matrix assumption still applies, weighted by the solid angles (see Figure 2). It is possible to prove this in some circumstances and we do so in Appendix A.

The convenient arrangement of materials in Figure 2 is unlikely to apply in a real case. However, in the absence of any other methodology we may assume that we can still use this method, approximating the solid angles that apply to each material as the volume occupied by that material in a sphere whose radius is the gamma attenuation depth and whose centre is at the sample, divided by the total volume of the sphere. The more complex the actual arrangement of materials, the more specious this approximation, as our results in Section 2.4 demonstrate. In the case of contexts with a simple arrangement of materials, this geometric approximation will frequently be acceptable (e.g., Reference [6]; see extended discussion in Reference [5]: 289). In the case of a complex archaeological context like a tomb, something more is needed.

##### DosiVox 

The large hadron collider (LHC) at CERN causes particles to collide with such high velocity that they break up into a shower of other particles, which are seen in the detector. Because the effects of these particles in the detector are indirect it is necessary to model the origin, transport and detection of these particles and then use statistical methods to determine the most likely sequence of events (e.g., Reference [7]).

One such model created and used at CERN is Geant-4, which simulates the creation, transport through matter and absorption of high-energy particles. Researchers at the Université Bordeaux Montaigne realised that this is exactly what was needed to model the dose rate that a luminescence sample experiences in its burial location, and thus the DosiVox software was developed [1].

As will be discussed further in Section 4.1, using DosiVox starts with making a model which includes the geospatial arrangement of all materials in the burial environment, as well as their composition. The composition of the materials includes their bulk composition, that is, the elements that make up the bulk of their mass (which affects the transport and absorption of the radiation) and the radioactive trace elements which emit the radiation (which are specified separately). We note that as well as specifying the composition and distribution of the materials, it is also possible to specify the grain-size of the sample; however, we omit this as we are only interested in the gamma radiation.

The dose absorbed by the sample location is the integral over time of the energy deposited by every gamma photon emitted from every radioisotope in the model. Since this integral is high-dimensional and expensive to calculate, DosiVox uses Monte-Carlo methods to approximate the integral, effectively allowing a given number of gamma particles to be generated at random from within the model and measuring the dose at the sample location from each particle. Thus, the greater the number of particles, the more accurate the calculation, as discussed in Section 4.2. For more details concerning the way DosiVox works the reader is referred to the original paper [1]. In this work, we wish to give a practical description of how DosiVox was applied, in the hopes that it may be useful to others wishing to use DosiVox in complex archaeological or geological contexts.

##### Other Gamma Sources

There are of course other sources of gamma radiation that might have affected the effective dose rate. Assuming that the sample was not exposed after excavation, the most notable of these is cosmic radiation. In our work here, we use the effective and well-established method of calculating the cosmic dose rate as a function of depth and global position of an archaeological/geological site that is provided by the Dose Rate and Age Calculator (DRAC) ([8]; following Reference [9]).

## 2. Results

### 2.1. Overview

To illustrate how DosiVox can be used to reconstruct radiation transport through complex burial environments, we look at two case studies from ancient Egypt. This research was initially carried out as part of Hood’s doctoral thesis, which produced the first ever OSL dates obtained for ancient Egyptian ceramics. The results of this dating programme can be found in References [10,11]. Owing to a current law in Egypt which prevents the removal of archaeological material for scientific analysis, this research was carried out on material excavated at the turn of the 20th century which was stored in museums outside of the country. As a result the burial environment and thus DosiVox model, was established through analysis of the original excavation reports, and when possible, more recent archaeological examinations (as was possible in one case, see below).

### 2.2. Case Study 1: Bêt Khallaf

The first case study looks at twenty-four ceramics sampled from the Upper Egyptian site of Bêt Khallaf. The site was excavated by British archaeologist John Garstang in the early 1900s, and the intact ceramic material recovered from the site was removed from Egypt soon after excavation and dispersed across three museums: the Ashmolean Museum in Oxford, the Garstang Museum of Archaeology in Liverpool and the University of Pennsylvania Museum of Archaeology and Anthropology in Pennsylvania (Penn Museum). The assemblage is typologically characteristic of the late Early Dynastic Period of Egypt (c. 2650 B.C.E.).

The Bêt Khallaf assemblage is a perfect candidate for testing and comparing gamma dose rates obtained using DosiVox to those determined using the IMA. This is firstly because many of the vessel types found in the tombs at Bêt Khallaf are diagnostic (thus providing a perfect platform for using absolute dating to anchor the relative ceramic typology to a known calendrical system), and secondly because Garstang kept and published reports of high standard for that era. These reports were used to construct the DosiVox model. In addition to providing details of the tomb architecture (tomb size, depth etc.), Garstang also gives the location of the pottery deposit within each tomb, allowing an accurate find spot of the ceramics to be determined (Reference [12]: 8–16).

### 2.3. Case Study 2: Tomb of Djer, Abydos

The second case study was carried out using four vessels from the Tomb of Djer at Abydos. Excavated by Flinders Petrie in the early 1900s, this Abydene tomb was the burial place of King Djer, the 3rd king of the first Egyptian dynasty (c. 3000 B.C.E.). After excavation, the ceramic material was transported to the Ashmolean Museum, Oxford. Similarly to the Bêt Khallaf material, the Djer material was well-documented for its time and Petrie discusses the finding of the vessels and their precise location within the tomb. The DosiVox model was constructed based upon Petrie’s excavation reports but was also supplemented by newly documented evidence from the tomb, which has recently been reexamined by the German Archaeological Institute. The combination of both sets of information allowed us to reconstruct the deposition of the vessels with a high degree of certainty and renders the Tomb of Djer a prime candidate for examining gamma dose rates and comparing the results obtained using DosiVox and the IMA.

### 2.4. Comparing Gamma Dose Rates

In both ancient Egyptian case studies, ${\dot{D}}_{ext}$ was determined using the U, Th and K concentrations from original depositional material found adhering to the vessels (under the rim and on a vessel handle). DosiVox was used to model the gamma dose rate received by the vessels from the surrounding burial environment (where the alpha and beta dose rates were determined using DRAC). The cosmic dose rate (${\dot{D}}_{cos}$) was determined by using information reconstructed using the original excavation reports and Google Earth (where one can easily identify the tombs from which the vessels came from and thus their elevation, etc). Using the archival material discussed above, it was possible to reconstruct the burial environment for the examined ceramics. In both cases DosiVox modelling was considered necessary as the depositional environment for both sites was considered complex, without straightforward sample geometry. Complex sample geometry in luminescence dating occurs when a sample is not obtained from a (more or less) homogeneous depositional environment. In contrast, a simple sample geometry is what we see in Figure 1.

In the case of Bêt Khallaf, the ceramics were not fully buried, but rather ‘strewn’ about in a presumably haphazard manner. The precise manner of their deposition is unknown (i.e., they could potentially be sitting upright, lying on their side, or halfway out of the tomb fill; as described in Section 4.2.1 these variables were tested to see their effect upon the determined gamma dose rate and have been presented below), but from the tomb reports we were confident that they were not fully buried and thus would be affected by complex sample geometry, consisting of the pot itself, the tomb fill/debris on the ground as well as the tomb walls.

In the case of the Tomb of Djer, Petrie recorded the exact find spot of these vessels in the tomb, and the unpublished work of the German Archaeological Institute further informed us that these vessels were positioned upright and buried up to their mid-point in the surrounding sediment of a chamber in the north west corner of the mud-brick tomb and covered by a mud-brick staircase. These vessels also contained contents which were a mixture of organic and inorganic material. All of these known details helped to reconstruct the complex sample geometry for these vessels and to make the resulting DosiVox model.

Through reconstruction of the burial environment our resulting DosiVox models were able to present a more realistic gamma dose rate measurement for the OSL sample than if we simply assumed 4π geometry or determined a ‘best guess’ geometry based on assumed percentage contributions from each material measured.

Table 1 and Table 2 present the calculated gamma dose rates for the Bêt Khallaf ceramics (Table 1) and those from the Tomb of Djer (Table 2). For each set of ceramics the gamma dose rate for each sample has been calculated in one of three ways: firstly by using a 4π geometric assumption using the IMA; secondly using a scaled IMA based on a ‘best guess’ of the complex sample geometry and; thirdly by using the gamma dose rate modelled using DosiVox. In the case of Bêt Khallaf, a sample ‘best guess’ of 2π is displayed. This was derived by following the principles outlined in Section 1.2.2, as the samples were known to be on the floor of the tomb (i.e., fill below and air above); in the case of the Tomb of Djer, the pots contained organic residue and were more than half buried in fill. Thus, a ‘best guess’ of 3π fill and 1π pot contents is displayed.

## 3. Discussion

We can see from the results presented in Table 1 and Table 2 that the gamma dose rates obtained by using DosiVox differ from those obtained by assuming ‘best guess’ scaled geometries. In the case of Bêt Khallaf the outcome saw DosiVox produce a gamma dose rate far more in keeping with an assumed 4π geometry than in our ‘best guess’ scaled geometry, illustrating how DosiVox can provide a result which is not subject to inbuilt biases and assumed geometries determined by the luminescence practitioner.

It can, of course, well be argued that the ‘best guess’ was in fact 4π, because the roof of the tomb was of similar composition to the floor and the dry air of the tomb would only slightly attenuate the radiation. We do not dispute this; we merely observe that all of these sweeping arguments and assumptions can be replaced by a more rigorous procedure using DosiVox. One benefit of this rigorous procedure is that there are many hidden assumptions in the 4π IMA estimate that DosiVox makes explicit and testable (position of the pot, etc). More importantly, however, it is only having run DosiVox (i.e., with hindsight) that we can know that the 4π IMA assumption was acceptable. Even though it might seem a reasonable assumption about the transport of gamma radiation *a priori*, with DosiVox the transport and absorption of gamma radiation is modelled in a way (using Geant-4) that has been extensively tested against highly accurate particle physics experiments; thus we can be much more confident in its results and we can eliminate at least one big assumption from our chain of reasoning.

In the case of the Tomb of Djer the DosiVox gamma dose rate was significantly different to the IMA and ‘best guess’ geometry dose rates. The complex depositional environment and the array of different elements affecting the dose rate was such that the gamma dose rate achieved using DosiVox modelling varied significantly enough from assumed geometries that final dose rate determinations varied considerably (which in turn affects the final luminescence age calculations). (It is beyond the scope of this paper to present the final age calculations for this material. If interested, the reader is referred to References [10,11], noting that final ages therein obtained using DosiVox were in good agreement with associated archaeological and historical chronologies.)

In simple geometric cases, for example, when a sample is known to have full, 4π geometry, DosiVox may be too significant an undertaking to apply to every luminescence sample and indeed will, in all likelihood, give a similar result. However, the more informative analysis and more accurate gamma dose rates achievable by DosiVox modelling in more complex burial environments, for example, that seen in the Tomb of Djer, illustrates how using the programme can improve luminescence age determination. In the case of complex sample geometries, it is therefore recommended that DosiVox modelling be incorporated into luminescence work to improve dose rate calculations and, in turn, age determinations.

One limitation of this research is that from the tomb reports we know that the ceramics at both sites were found as caches, that is multiple vessels were found in the same deposit. However, it was impossible at either site to determine the order or precise location of the ceramics relative to one another. In an ideal situation, if this information were recorded at the time of excavation, it would be possible to improve upon the DosiVox model even further by adding in each individual ceramic into the model so that the gamma dose rate determined for each vessel would also factor in the gamma dose being emitted by the surrounding vessels. This could be easily achievable for those wanting to use DosiVox modelling of complex deposits found in current excavations or fieldwork situations.

DosiVox is a valuable new tool when dealing with samples that have complex geometry and where in situ gamma measurements are not able to be made. Using DosiVox in conjunction with current projects could yield gamma dose rates far more accurate than those achievable through a simple incorporation of the IMA. This is because, in most cases, all the elements required to construct a DosiVox model would be readily available and, as a result of the more detailed model and more limited (and explicit) assumptions, a higher degree of confidence could be placed upon such a model, thus potentially yielding more accurate gamma dose rates than the IMA alone. We recommend that DosiVox modelling is routinely carried out at locations where in situ measurements are not available.

DosiVox modelling can be used in both helping to determine the gamma dose rate at recently excavated sites, those sites excavated in the past (i.e., where the resulting material has been stored in museums) and for both geological and archaeological situations. We are of the opinion that the incorporation of DosiVox into luminescence research can be of significant value in a number of situations, and believe that more accurate gamma dose rates can be achieved using DosiVox in cases of complex sample geometry. As such, DosiVox has the ability to greatly improve the reconstruction of dating profiles at geological and archaeological sites worldwide and assist in more accurate chronologies being achieved.

## 4. Materials and Methods

### 4.1. Constructing a DosiVox Model

A DosiVox model broadly consists of two parts:

1. a model of the composition and layout of the materials in the burial environment, and
2. a similar but more detailed model of the target site (the place where the sample is drawn from).

Before discussing how those parts are created it is helpful define the following terms:

- *Material*—the model is divided into regions that are filled with materials that are broadly uniform in composition: for example, a layer of sand, or a mud-brick wall.
- *Component*—materials are described by their density, their water content and the relative quantities of their chemical components (e.g., SiO_2_ or TiO).
- *Voxel*—the whole burial environment is broken up into a grid of cubes which are called voxels (effectively “3D pixels”). Each voxel contains one material.
- *Subvoxel*—in the mode of DosiVox used in this paper, one voxel in the model may be broken down into a fine grid made up of subvoxels. This voxel is referred to as the ‘sub-voxelized voxel’. The sub-voxelized voxel is used when one part of the model needs to be described in more detail. In this work, we have the case of a ceramic vessel in a tomb. The tomb itself is described at lower resolution using the voxels; one of these voxels (the sub-voxelized voxel) contains the vessel, which is described at higher resolution using subvoxels. This is elaborated below in Section 4.1.1.

#### 4.1.1. The Model Layout

The volume that is being modelled (e.g., a tomb) must first be reconstructed, and then represented in terms of a 3D regular grid of voxels. Reconstructing the volume may be done using anything from field reports and excavation maps, to the latest 3D mapping tools like *GIS*. Here we discuss our own work using excavation reports from the early 20th century.

As discussed above, a major benefit of the Bêt Khallaf and Djer material is that Garstang’s and Petrie’s excavation records [12,13] were of excellent quality for the early 20th century and they allow us to reconstruct with a reasonable degree of confidence the exact burial environment of each vessel, which is further strengthened by finding parallels for similar temporal contexts and recent excavations (i.e., in the case of the Tomb of Djer).

Regardless of the source of information about the burial environment, what must eventually be constructed is a series of evenly spaced layers (i.e., horizontal sections) each of which is composed of an identical regular grid of voxels. In our model, the voxels were sized 30 cm × 30cm × 15 cm, with the total number of voxels varying with the size of the tomb being modelled. Thus, once the tomb shapes and sizes were reconstructed, each tomb layout was replicated using voxels in a three-dimensional grid.

Every voxel interior in the grid must be labelled with a number identifying a particular material. One of the voxels on one of the layers must be chosen as the sub-voxelized voxel—in our case, the location of the vessel of interest (as far as could be known, as we discuss below). Within the sub-voxelized voxel the whole process is repeated again on a finer scale. The voxel is divided into layers, which are divided into rectangular cells. Each cell, that is, each *subvoxel*, is labelled by a material representing a number (this process allows a higher spatial resolution in a given section of the tomb (i.e., the section that contains the vessel, without the cost of having such a high spatial resolution across the whole tomb ([14])).

A variety of possibilities are available when constructing the sub-voxelized voxel. As discussed in Reference [14], there are many 3D scanning tools which may be used to make a 3D image of the vessel, which can be converted into a series of subvoxel layers by, for example, associating colours with materials. In our work, we constructed the sub-voxelized voxel manually from drawings of the vessels. While we were careful to preserve the overall scale and thickness of the vessel, we determined it was unnecessary to precisely specify the shape of the vessel, owing to the long attenuation depth of the gamma rays. Accordingly, each jar-shaped vessel was simulated using a simplified model of the vessel composed of 1 cm × 1 cm × 1 cm sub-voxels forming a regular rectangular shape (and for the bowls and the pot stand other approximate shapes were used).

**Figure 3:**
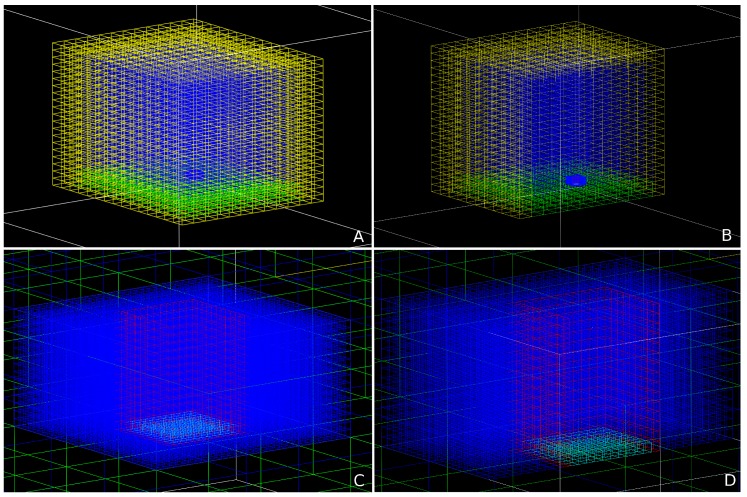
Wire frame representations of the DosiVox model for X5472. The whole tomb is subdivided into voxels which are filled with different components: yellow = gebel/bedrock, green = fill, blue = air, red = vessel walls, cyan = vessel base where the dose is recorded. (**A**–**D**) represent different views of the model: (**A**) whole tomb; (**B**) whole tomb cut away to reveal the detector (the sub-voxelised voxel which contains the vessel); (**C**) close up view of the detector, (**D**) detector cut away to reveal the vessel. NB these figures represent an unburied vessel.

**Figure 4:**
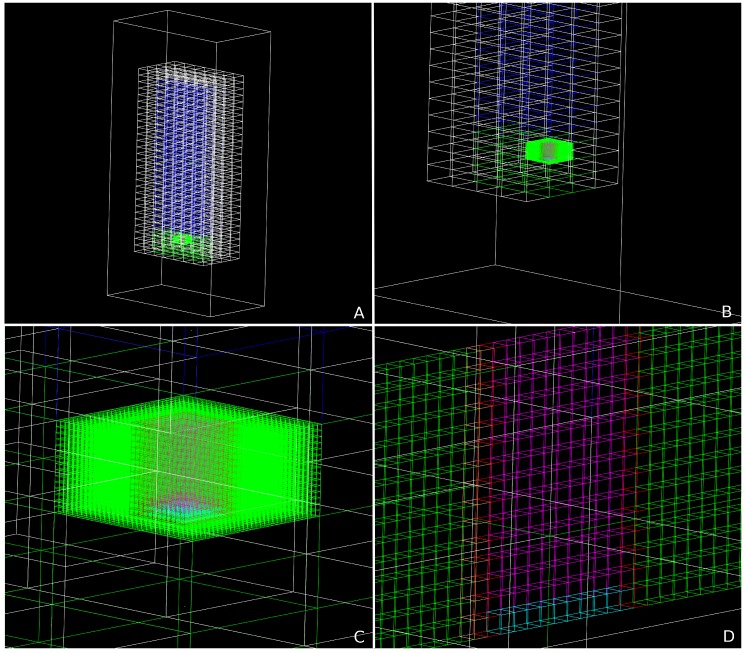
Wire frame representations of the DosiVox model for X5476 in the Tomb of Djer. The whole tomb is subdivided into voxels which are filled with different components: white = mud-brick, green = fill, blue = air, red = vessel walls, cyan = vessel base, orange = sediment attached to vessel wall (seen in (**D**) only), magenta = pot contents/residue. (**A**–**D**) represent different views of the model: (**A**) whole tomb; (**B**) whole tomb cut away to reveal the detector (the sub-voxelised voxel which contains the vessel); (**C**) close up view of the detector, (**D**) detector cut away to reveal a cross-section of the vessel and surrounding fill.

Figure 3 presents a set of schematic representations of the DosiVox simulation model used for vessel X5472 in tomb K2 at Bêt Khallaf. Figure 4 presents a similar set for vessel X5476 in the tomb of Djer. Supplementary Materials Video S1 presents a representative 3D visualisation of a ceramic vessel in a tomb.

#### 4.1.2. Describing Materials

Having labelled all voxels and subvoxels as containing a particular material, we now need to define these materials. For example, the materials of the tombs at Bêt Khallaf included air (within the tomb chamber), a sand-based fill (on the floor of the tomb), and gebel—Egyptian bedrock—which comprised the walls and roof of the tomb.

To define a material, we require its density, its bulk chemical composition, the concentrations of radioisotopes, and the water content. As with the tomb dimensions, the methodology for determining this information for each material will vary depending on the project. In an ideal situation, a large sample would be obtained of each material, which would then be sent away for analysis using, for example, a form of mass spectrometry. However, in most cases, all that is possible is to incorporate all available information, and to make assumptions where necessary. The assumptions themselves can then be tested, as we demonstrate below.

Below we present a complete list of all the materials used in this project, the methods used to analyse them and necessary assumptions.

- *Air*—assumed to be typical atmospheric concentration, with water content based on a specified temperature.
- *Fill at Bêt Khallaf*—bulk chemical composition and radioisotopes determined from analysis of sediment attached to the outside of the vessels; density assumed to be that of loose sand.
- *Gebel at Bêt Khallaf*—chemical composition and water content assumed to be the same as the fill; density assumed to be that of sandstone;
- *Fill at Djer*—radioisotopes determined from analysis of sediment attached to the outside of one vessel; bulk concentrations assumed to be similar to those at Bêt Khallaf;
- *Clay (vessel fabric)*—measurement of the bulk composition is discussed below in Appendix B.1; radioisotope concentrations were measured separately for every vessel using ICP-MS analysis of samples extracted using the minimum extraction technique [15] (the sub-voxels within the vessel were classified as two separate materials—‘Clay’ and ‘ClayBase’—so that the average dose rate was recorded for the base (i.e., the OSL sample location) and the rest of the vessel separately. However, these two clay sub-voxel types were identical in their composition).
- *Pot contents at Bêt Khallaf*—vessels at Bêt Khallaf were assumed to be half-filled with fill.
- *Pot contents in the Tomb of Djer*—the vessels in the Tomb of Djer contained the organic/minerogenic residue of their original contents; this residue had been analysed as part of another project [11,16,17].
- *Mud-brick walls in the Tomb of Djer*—no direct samples or measurements were available of the mud-brick composition. Accordingly, the composition of the mud-brick was assumed to be similar to that of Nile silt clay.

The full chemical breakdowns of each material that resulted from these analyses and assumptions are detailed in Appendix B.

#### 4.1.3. Specifying Radioisotope Concentrations

While the bulk chemical composition is specified only once per material, the radioisotope concentration is specified on a per-voxel (or per-subvoxel) basis. This allows the same material to have different radioisotope concentrations in different places. Accordingly, as well as a grid specifying which material is in which voxel (and another for subvoxels), we must also provide grids specifying the radioisotope concentration in each voxel/subvoxel. DosiVox calculates the dose from each radioactive element separately, and so a separate grid must be provided for each one.

### 4.2. Running and Resolving DosiVox

Once all the necessary data is assembled, it is time to input that data into DosiVox and then use it to calculate the dose rates. Running DosiVox consists of the following steps.

1. Obtaining DosiVox.
2. Generating a DosiVox pilot file.
3. Running DosiVox.
4. Extracting the dose rates for each voxel and subvoxel.
5. Repeating the previous two steps with larger and larger numbers of particles, up to the point where the dose rate is known to the required precision.

For the first step readers are referred to the DosiVox documentation [14]. For carrying out the second step, DosiVox provides a graphical tool. However it is also perfectly possible to manually construct a pilot file based on the included files. In this work, a template pilot file was manually constructed for each sample studied. From that point, an automated tool called CodeRunner [18,19] was used to

1. set the radioisotope concentrations in the pilot file (for U, Th and K in turn);
2. run steps 3 and 4 above repeatedly, increasing the number of particles until the dose rates converged (that is, until the dose rates are known to the required precision);
3. parallelize the process by running multiple identical simulations concurrently, as detailed towards the end of this section.

Because of the Monte-Carlo methods used by DosiVox, there is no concept of time in the simulation. What is available in the DosiVox output data is the total dose emitted ($E_{\gamma }$) and the total dose absorbed ($D_{\gamma }$) by each voxel and subvoxel during the course of the simulation. In order to convert that total absorbed dose into a dose *rate* (${\dot{D}}_{\gamma }$), we must scale it using the emitted dose rate ${\dot{E}}_{\gamma }$ (a known quantity given the concentration of the particular radioisotope, obtainable from, for example, Reference [4]). Thus, the absorbed dose rate from a given voxel or subvoxel is

(1) $${\dot{D}}_{\gamma }=\frac{D_{\gamma }}{E_{\gamma }}{\dot{E}}_{\gamma }.$$

This conversion was carried out automatically by the CodeRunner tool.

The output $D_{\gamma }$ is provided voxel by voxel and subvoxel by subvoxel. However, it is also provided as an average per material. As mentioned earlier in Section 4.1.2, we define the material of the base of the vessel (where the samples were taken in every case) separately to the walls. Thus in every case we are calculating the average dose rate in the base of the vessel.

Given the complexity of the model and the relatively small volume occupied by the target site (the base of the vessel), a very large number of particles were required to achieve a sufficiently low uncertainty in the dose rates. Since the simulations were carried out on a laptop (albeit one with an up-to-date hyperthreaded 4-core CPU) total CPU time was limited. Nonetheless, even with the limited simulation time available, almost 550 simulations were carried out for this project (using approximately 5000 CPU hours). High-resolution simulations used 8 million particles; lower-resolution simulations used 2 or 4 million particles. For each case, six individual simulations were run in parallel (i.e., the same case, and hence the same input file, was run in parallel). As DosiVox uses a Monte Carlo method it was possible to take the mean of the six simulations and combine these to increase the simulation resolution to 48 million particles per high-resolution case, that is, six parallel simulations × 8 million particles (Pers. Comm. L. Martin, 2015).

#### 4.2.1. Testing Assumptions in the Reconstruction

As touched on above, the information provided about the burial environments were incomplete, and several assumptions had to be made when constructing the DosiVox models. A DosiVox model allows us to obtain the best possible estimates given available sources of information. However, importantly, it allows us to assess the size of the error that may result from these assumptions. Thus, DosiVox allows us to be quantitative about the effect of missing information from the archaeological or geological record.

In this section we detail several of the necessary assumptions that were made for this project, and assess their impact on the accuracy of our results. These are assumptions concerned with:

1. The orientation of the vessel: the pot can be either standing (upright or upside down), or lying on its side. Since the OSL sample is drilled from the base in all instances in this project, the orientation of the pot in its burial environment will affect ${\dot{D}}_{\gamma }$ (i.e., was the sample location submerged in fill or exposed to air?)
2. Whether standing or lying on its side, the pot can be buried from 0% to 100% in fill, with the remainder surrounded by air (or other material).
3. The vessels can be filled from 0% to 100% with either fill or other contents (e.g., organic bulk residue).
4. The density of the vessel’s clay matrix.
5. The density of the fill.
6. The vessel’s proximity to the tomb walls, that is, its location within the tomb. Was it in the centre, corner, or along a side of the tomb?
7. The moisture content of the burial environment.

**Figure 5:**
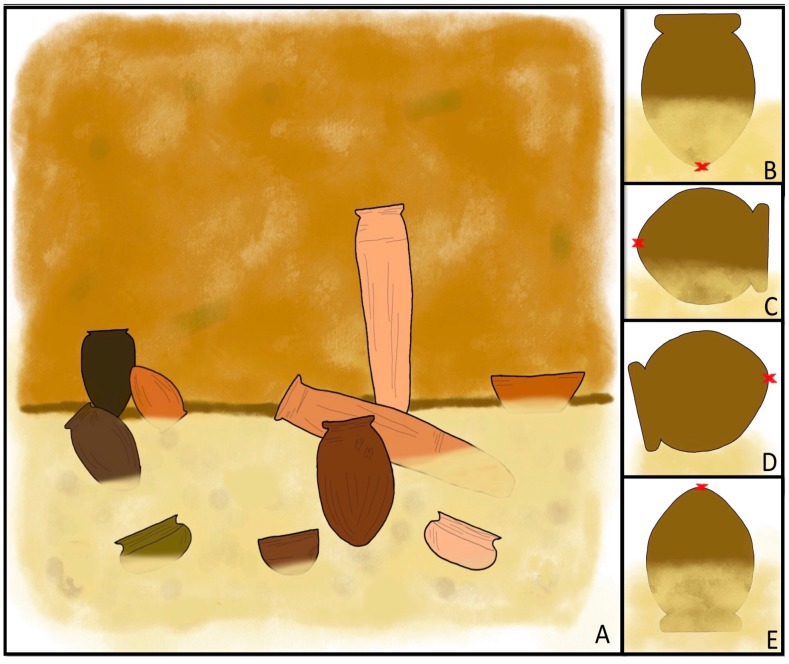
(**A**–**E**): A: schematic representation of a ceramic cache in a chamber tomb; (**B**–**E**): the four most common ways in which a ceramic vessel is deposited in a cache—the vessel is standing upright in the fill (**B**), the vessel is lying on its side partially submerged in fill (**C**), the vessel is lying on its side and resting on top of the fill (**D**), or the vessel is upside down in the fill (**E**). The red crosses seen in (**B**–**E**) are illustrative of OSL sample locations as it is considered best practice to take these samples from the base of a vessel wherever possible.

With regard to the position and burial situation of the vessels found within chamber tombs (i.e., where the tomb is not entirely filled up with fill, such as in the cases of Bêt Khallaf and the Tomb of Djer), parallels at recently excavated sites can help inform of the most likely way in which the vessels were found buried. Figure 5A provides a schematic representation of how a ceramic vessel cache may look in an Early Dynastic chamber tomb (based on Hood’s personal observations in the field). As can be seen, the vessels in such caches can be positioned in a number of ways and Figure 5B–E show the four most likely of these.

Owing to time constraints caused by the significant simulation time needed to test each case, this project limited itself to testing the first five assumptions listed above at high resolution, as these were deemed the most likely to affect ${\dot{D}}_{ext}$. Of the final two assumptions (assumptions 6–7), assumption 6 was considered, but at low resolution (the attenuation of radiation through air with low moisture content is such that it should not matter where the vessel is located as it will, in theory, still receive radiation from all four walls of the tomb). Assumption 7 was not tested as the literature consulted seemed unanimous that the correct approximate value for water content within the burial environment was 3% ± 2% ([20,21,22,23]). One situation that can not be tested for in these case studies is the proximity of other vessels to the case vessel. It was impossible, for lack of information, to determine the order or arrangement in which the vessels were located in the tombs. Thus, each vessel was modelled to be the only vessel in the modelled tomb (see Section 3).

**Figure 6:**
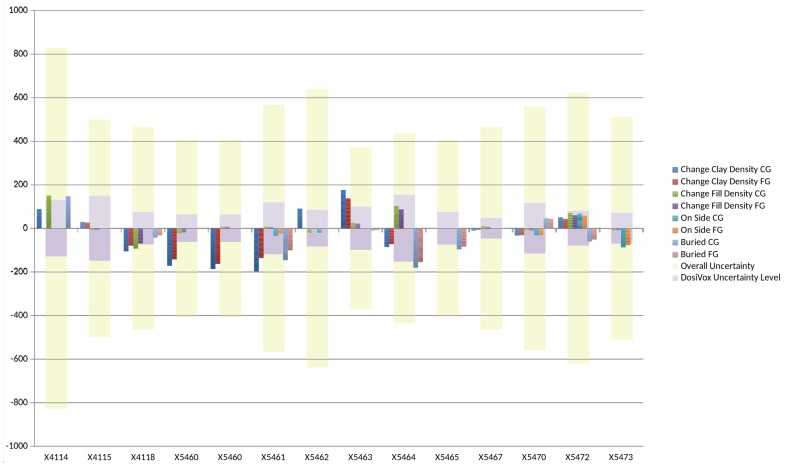
Graph depicting the effects of changes to the assumptions made in the DosiVox modelling. The change in the final age in years as a result of changing each assumption is plotted (using internal dose rate and equivalent dose rate data from Reference [10]). For each assumption we change, we display the effect on the age as determined from the coarse grain fraction (CG) and the fine grain fraction (FG) from the sample (NB a final age would result from combining these two values). The inner set of larger bars represent the magnitude of the error in years (at the 1σ level) resulting from the uncertainty in the DosiVox result for the reference case. It can be seen that changing all assumptions results only in errors which are comparable to or less than the typical error in the DosiVox simulations (which is itself small compared to the overall error). This means that there is insufficient evidence to reject the null hypothesis (the null hypothesis being that there is no effect of changes, or more precisely, that the effect of changes is smaller in magnitude than the uncertainty resulting from the DosiVox simulations). The outer set of larger bars represents the magnitude of overall uncertainty of the measured sample age in years. We can see that the DosiVox-derived uncertainty is itself only a minor contribution to the overall uncertainty, and that since the null hypothesis states that the effect of changes in assumptions are small, i.e., undetectable, with respect to the DosiVox-derived uncertainty, we can therefore conclude that *the effect of changing the assumptions is negligible*.

A full description of the tests carried out for these seven assumptions and the effect of their changes on ${\dot{D}}_{\gamma }$ is given in Table 3 and illustrated in Figure 6.

Overall it was found that changing these assumptions only had a weak effect (i.e., if there was an effect its magnitude was not statistically significant). This somewhat unexpected result is certainly worthy of further study. It is likely to be a consequence of the particular circumstances being considered here (specific dosimetries and so on). However, it may be hypothesised that it could be due to the characteristics of the emission spectra of the radionuclides. The emission spectra of U and Th are dominated by lower-energy, short-range gamma rays. Since the concentrations of U and Th are larger in the clay than in the surrounding fill, it might be expected that the dose rate from these radionuclides is dominated by the dose rate coming from the clay of the vessel (effectively the internal gamma dose rate) and will thus be unaffected by most of the assumptions tested here. By contrast the K spectrum is dominated by one high-energy, long-range gamma emission, for which the attenuation rate in any of the components of the model of the tomb will be low. Therefore, whatever the orientation of the vessel or its immediate surroundings, the absorbed K dose rate will effectively be an average of the tomb as a whole, i.e., it will be affected by whatever material is within 30 cm of it, or 30 cm of the material which is separated from the pot by air (as the gamma radiation will travel directly through air with low moisture content). Thus, even though the incomplete archaeological literature produced unavoidable uncertainty in the model, this did not give rise to significant uncertainty in the calculated ${\dot{D}}_{\gamma }$.

## Figures and Tables

**Table 1:** A comparison of the DosiVox gamma dose rate and the gamma geometries (full 4π geometry and a ‘best guess’ scaled geometry of 2π, obtained using DRAC) for the tombs at Bêt Khallaf.

Sample ID	DRAC ${\dot{D}}_{\gamma }$ (4π Geometry) (Gy ka${}^{-1}$)	DRAC ${\dot{D}}_{\gamma }$ Error (4π Geometry) (Gy ka${}^{-1}$)	DRAC ${\dot{D}}_{\gamma }$ (2π Geometry) (Gy ka${}^{-1}$)	DRAC ${\dot{D}}_{\gamma }$ Error (2π Geometry) (Gy ka${}^{-1}$)	DosiVox ${\dot{D}}_{\gamma }$ (Gy ka${}^{-1}$)	DosiVox ${\dot{D}}_{\gamma }$ Error (Gy ka${}^{-1}$)
X4114	0.523	0.033	0.262	0.017	0.507	0.099
X4115	0.523	0.033	0.262	0.017	0.600	0.132
X4116	0.523	0.033	0.262	0.017	0.582	0.091
X4117	0.523	0.033	0.262	0.017	0.575	0.087
X4118	0.523	0.033	0.262	0.017	0.551	0.088
X5458	0.600	0.039	0.300	0.020	0.546	0.107
X5459	0.459	0.029	0.230	0.015	0.556	0.085
X5460	0.523	0.033	0.262	0.017	0.526	0.094
X5461	0.523	0.033	0.262	0.017	0.591	0.121
X5462	0.523	0.033	0.262	0.017	0.565	0.090
X5463	0.619	0.039	0.310	0.020	0.560	0.086
X5464	0.523	0.033	0.262	0.017	0.524	0.100
X5465	0.523	0.033	0.262	0.017	0.559	0.089
X5466	0.523	0.033	0.262	0.017	0.638	0.109
X5467	0.523	0.033	0.262	0.017	0.573	0.091
X5468	0.523	0.033	0.262	0.017	0.564	0.090
X5470	0.523	0.033	0.262	0.017	0.538	0.102
X5472	0.523	0.033	0.262	0.017	0.552	0.096
X5473	0.523	0.033	0.262	0.017	0.537	0.087

**Table 2:** A comparison of the DosiVox gamma dose rate and the gamma geometries (full 4π geometry and a ‘best guess’ scaled geometry of 3π + 1π, obtained using DRAC), for the Tomb of Djer.

Sample ID	DRAC ${\dot{D}}_{\gamma }$ (4π Geometry) (Gy ka${}^{-1}$)	DRAC ${\dot{D}}_{\gamma }$ Error (4π Geometry) (Gy ka${}^{-1}$)	DRAC ${\dot{D}}_{\gamma }$ (Scaled Geometry) (Gy ka${}^{-1}$)	DRAC ${\dot{D}}_{\gamma }$ Error (Scaled Geometry) (Gy ka${}^{-1}$)	DosiVox ${\dot{D}}_{\gamma }$ (Gy ka${}^{-1}$)	DosiVox ${\dot{D}}_{\gamma }$ Error (Gy ka${}^{-1}$)
X5477	0.174	0.012	0.212	0.01	0.15	0.02
X5478	0.174	0.012	0.212	0.01	0.452	0.06
X5479	0.174	0.012	0.212	0.01	0.423	0.06
X6114	0.458	0.029	0.229	0.015	0.504	0.07
X6115	0.324	0.021	0.162	0.011	0.469	0.06
X6116	0.439	0.028	0.220	0.014	0.496	0.06
X6120	0.174	0.012	0.158	0.008	0.489	0.08

**Table 3:** The assumptions tested by DosiVox, illustrating the possible scenarios/assumptions that could be made (left column) and the outcome of the DosiVox modelling for each assumption (right column).

Modelling Unknown	Effect on Modelled Dose Rate of the Bêt Khallaf Assemblage
1. The pot can be (1) standing (upright or upside down), or (2) lying on its side. Since the OSL sample is drilled from the base in all instances, it also matters whether the sample location was on the upper or lower side of the deposited vessel (i.e., was the sample location submerged in fill or exposed to air)?	Two scenarios were considered: the pot standing on its base or lying on its side. These were considered the two limiting cases and as no measurable change was observed, it was concluded that the orientation of the pot and the location of the sample had no significant effect. To further examine the seeming minimal effect of vessel orientation on the measured ${\dot{D}}_{ext}$, it would be possible in the future to determine ${\dot{D}}_{ext}$ for a set of samples taken from different locations on the same vessel.
2. Whether standing or lying on its side, the pot can be buried up to 100% in fill, or be unburied and thus exposed to air up to 100%.	Two scenarios were considered: the vessel lying on the surface of the fill or buried ∼15 cm, which for bowls means they are completely buried and for storage jars, they are buried up to half their height. Again, no measurable change was observed.
3. The vessels can be filled from 0% to 100% with either fill or other contents (e.g., organic bulk residue).	Two scenarios were considered: the vessel was filled with fill or it had no fill inside it. Again, no measurable change was observed.
4. How does the density of the vessel’s clay matrix affect the external $\dot{D}$?	Although we were not able to measure the clay density of all vessels, a single sherd (X5460) was measured as having a density of 1.4 g/cm${}^{3}$. In the visual analysis of the clay fabric that accompanied each of the vessels the density of the vessels was determined as being ‘high’, ‘medium’, or ‘low’. Independently of the density measurement carried out upon X5460, the vessel this sherd came from was deemed to have a ‘low’ density. Thus, based on this information, we were able to extrapolate that the density for vessels deemed to have a medium density was likely to be ∼1.6 g/cm${}^{3}$, and for vessels with a ‘high’ density, ∼1.8 g/cm${}^{3}$.Two scenarios were considered—the clay density was either set to its known value or to a reference value of 1.8 g/cm${}^{3}$. No measurable change was observed between the two scenarios and thus it was concluded that although the clay densities are not known precisely for each vessel, variation within the likely range of values will not affect the result.
5. How does the density of the fill affect the external $\dot{D}$?	Two different fill densities were considered—1.2 g/cm${}^{3}$ and 1.7 g/cm${}^{3}$ and no measurable change was observed. Thus while the density of the fill is an unknown quantity, given that the fill density is visibly less than that of the low density clay whose density was measured (as above), the value of 1.2 g/cm${}^{3}$ was used in final dose rate calculation. Thus, while the exact density of the Bêt Khallaf tomb fill can not be reconstructed accurately, simulation of two different scenarios illustrates that within the likely range of fill densities which could be present at Bêt Khallaf, variation within this range will not affect the modelled dose rate.
6. The vessel’s proximity to the tomb walls, that is, its location within the tomb. Was it in the centre, corner, or along a side of the tomb?	A limited study of this was carried out at low resolution and no measurable changes were observed. Given the very low attenuation rates of gamma rays in air with low moisture content, this is to be expected.

**Figure A1:**
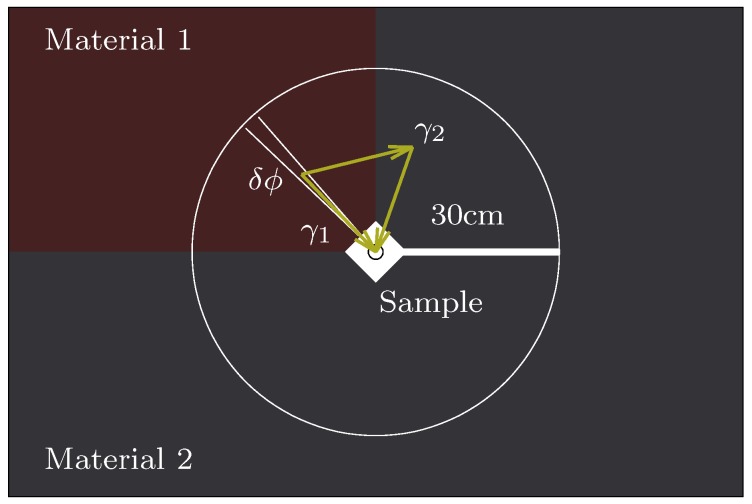
A sample surrounded by two materials; a solid angle δϕ is projected onto the 2d plane; the small black circle represents an infinitesimal volume δV; gamma photons may arrive in δV directly ($\gamma_{1}$) or after one or more intermediate collisions ($\gamma_{2}$).

## Supplementary Materials

The following are available online at https://www.mdpi.com/2409-9279/2/4/91/s1, Video S1: 3D expanded view of a DosiVox model.

## Abbreviations

The following abbreviations are used in this manuscript:

OSL	Optically stimulated luminescence
IMA	Infinite matrix assumption
DRAC	Dose Rate and Age Calculator

## Appendix A. Infinite Minute Matrix Assumption Plus Geometric Assumption

We wish to determine the radiation absorbed per unit time in an infinitesimal volume δV in the situation illustrated in Figure A1. In the case where Material 2 is identical to Material 1, the infinite matrix assumption (IMA) applies and the radiation absorbed is equal to $E_{\gamma 1}\delta V$ where $E_{\gamma 1}$ is the volumetric emissivity of Material 1. However, we may also write it as

(A1) $$E_{\gamma 1}\delta V=\int_{0}^{30}\delta r\int \delta \phi r^{2}\Gamma \left(r,\phi \right),$$

where *r* is the radius and Γ(r,ϕ) is the dose rate in δV that originates at radius *r* and solid angle ϕ.

Now let us consider the case where Material 2 is not identical to Material 1. Let us first define $\Gamma_{1}^{a}\left(r,\phi \right)$ to be the dose rate from material *a* where only one collision occurs (equivalent to path $\gamma_{1}$ in Figure A1). If we can neglect all intermediate (Compton) collisions, then we can write down the dose rate in δV as

(A2) $$\int_{0}^{30}\delta r\int_{\mathrm{Material}1}\delta \phi r^{2}\Gamma_{1}^{1}\left(r,\phi \right),+\int_{0}^{30}\delta r\int_{\mathrm{Material}2}\delta \phi r^{2}\Gamma_{1}^{2}\left(r,\phi \right)=f_{1}E_{\gamma 1}\delta V+f_{2}E_{\gamma 2}\delta V$$

where $f_{1}$ and $f_{2}$ are the fractions of the total volume of the white sphere occupied by Materials 1 and 2 respectively. The right hand side of Equation (A2) is precisely the geometric approximation discussed in Section 1.2.2.

What about if intermediate collisions cannot be neglected? In fact the direct path $\gamma_{1}$ typically accounts for only half the dose (Ref. [5]: 289). In this case the geometric assumption still holds approximately for Figure A1 provided the attenuation of gamma radiation is similar for Materials 1 and 2. This is likely to be the case if, for example, Materials 1 and 2 have similar bulk compositions, differing chiefly in their radioisotope profiles. If, on the other hand, the materials have different attenuation properties, then the geometric assumption is not expected to apply.

Lastly, we note that in the case where the sample does not lie at the interface between two materials, one may use approximations like those described in Ref. [5]: 291.

## Appendix B. Determining Chemical Compositions

### Appendix B.1. The Bulk Composition of the Clays

The composition data for the Nile silt clay was provided by five bulk samples initially tested by a commercial laboratory, which in addition to the elemental analysis, provided a breakdown of the main components of the clay in percentages. With only five samples analysed it was not possible to know the specific chemical breakdown of each individual vessel from the Bêt Khallaf assemblage. Instead, based on these five samples, an average breakdown was determined (See Table A1). Table A2 gives the chemical composition breakdown of marl clay and Nile silt clay used in the DosiVox simulations (for more detailed discussion see Ref. [11]).

**Table A1:** Chemical breakdown of five ceramic vessels from Bêt Khallaf obtained using ICP-MS.

Sample	SiO_2_	Al_2_O_3_	Fe_2_O_3_	MnO	MgO	CaO	Na_2_O	K_2_O	TiO_2_	P_2_O_5_	Vacuum	Total
X5460	57.62	13.01	8.2	0.124	2.49	3.58	2.11	2.2	1.52	0.6	8.54	91.46
X4112	51.49	14.6	8.5	0.251	1.87	6.07	1.41	1.62	1.661	0.64	11.89	88.11
X4114	67.02	11.98	8.17	0.209	2.26	3.54	1.96	1.46	1.524	0.52	1.36	98.64
X4118	61.49	11.96	7.9	0.131	2.4	4.68	2.18	1.85	1.587	0.72	5.09	94.91
X4119	65.15	11.99	7.79	0.123	2.11	3.53	1.7	1.86	1.462	0.56	3.74	96.26
Mean Value	60.55	12.71	8.11	0.17	2.23	4.28	1.87	1.80	1.55	0.61	6.12	93.88

**Table A2:** Chemical breakdown of Nile clay, take from Table A1, and marl clay, taken from [24], used in the DosiVox simulations.

Clay	SiO_2_	Al_2_O_3_	Fe_2_O_3_	MnO	MgO	CaO	Na_2_O	K_2_O	TiO_2_	P_2_O_5_	Vacuum	Total
Nile	60.55	12.71	8.11	0.17	2.23	4.28	1.87	1.80	1.55	0.61	6.12	93.88
marl	41.3	21.95	6.76	0.05	1.8	18.8	1.39	1.20	0.90	0.45	1.3	98.7

### Appendix B.2. Determining Radioisotope Concentrations

#### Appendix B.2.1. Isotope Concentrations in the Clay and Pot Contents

The radioisotope concentrations in the clay of the vessels were also needed to calculate the internal dose rate, and thus were already available for inclusion in the DosiVox model. Owing to the samples being taken from museum specimens, the minimum extraction technique was used for nearly all sample collection [15]. The first ∼½ of the sample was analysed using ICP-MS. Special care was taken with sample collection and measurement owing to the small size of the sample [11,15].

Table A3 and Table A4 give the measured concentrations of radioisotopes in the clay and (in the case of the Tomb of Djer) contents of the vessels from Bêt Khallaf and Tomb of Djer respectively.

**Table A3:** Concentrations of U, Th and K (to three significant figures) for each Bêt Khallaf ceramic sample, measured using ICP-MS analysis, used to determine ${\dot{D}}_{int}$.

Sample	K (%)	^232^Th (ppm)	^238^U (ppm)
X4114	0.987	4.32	1.03
X4115	1.26	5.82	1.44
X4116	0.977	7.30	1.99
X4117	1.25	7.49	1.85
X4118	1.42	5.79	1.48
X5458	0.972	6.70	1.82
X5459	1.01	7.69	2.45
X5460	1.42	5.79	1.48
X5461	1.51	5.56	1.31
X5462	1.32	4.90	1.19
X5463	1.45	5.95	1.24
X5464	1.33	5.78	1.27
X5465	1.41	7.06	1.70
X5466	1.59	17.6	3.68
X5467	1.48	5.63	1.15
X5468	1.04	6.95	1.88
X5470	1.27	6.05	1.14
X5472	1.37	5.15	1.43
X5473	1.22	5.64	1.77

**Table A4:** Concentrations of U, Th, and K for each sample from the Tomb of Djer, measured using ICP-MS analysis, used to determine ${\dot{D}}_{int}$. Samples beginning with X5 are the clay fabric of the vessel; samples beginning with X6 are pot contents from select vessels.

Sample	K (%)	^232^Th (ppm)	^238^U (ppm)
X5474	0.87	8.15	3.75
X5475	1.08	9.03	4.21
X5476	1.21	14.19	3.39
X5477	1.00	10.57	1.93
X5478	0.85	8.75	3.46
X5479	1.00	7.99	3.20
X6114	0.51	4.2	1.3
X6115	0.37	3.2	0.8
X6116	0.38	3.3	1.8
X6120	0.13	2.26	0.35

#### Appendix B.2.2. Radioisotope Concentrations in the Fill

For the Bêt Khallaf material, it was noticed during the sampling process that several vessels had material adhering to their interior surface (vessels X5458, X5458, X5463, X5467, and X4120), or lodged tightly up under the rim of the vessel (vessels X5458 and X5459). It was possible therefore to collect this material for ICP-MS analysis and use the radionuclide concentrations as a proxy for the entire assemblage (see Table A5).

**Table A5:** Concentrations of radioisotopes in sediment samples in direct association with several Bêt Khallaf ceramic vessels.

Sample	Location of Sample	Concentration of K (%)	Concentration of ^232^Th (ppm) ^1^	Concentration of ^238^U (ppm)
X5458	Interior of vessel	1.38	4.4	1.3
X5459	Interior of vessel	0.88	3.9	1.5
X5463	Interior of vessel	0.88	4.6	1.8
X5467	Interior of vessel	0.41	1.38	0.45
X4120	Interior of vessel	0.81	3.2	2
X5458	Under rim	0.98	3.4	1.2
X5459	Under rim	0.71	3.2	1.4
Average		0.86 ± 0.11	3.44 ± 0.40	1.38 ± 0.19

^1^ ppm = parts per million mass.

The ICP-MS values for this project that were used to determine ${\dot{D}}_{ext}$ are presented in Table A5, with the average value being used. It was decided that an average value would be the best value to use for ${\dot{D}}_{ext}$ calculation, even though the values varied slightly between the individual vessels yielding external sediment. This was because the long attenuation depth of the external gamma radiation means that ${\dot{D}}_{ext}$ is affected by a larger body of material than the sediment in direct contact with the vessel. In this case, an average value will be more representative than an individual measurement as it will compensate for the variation in composition of the surrounding sediment.
